# Epithelioid Hemangioendothelioma of Mandibular Gingiva: A Challenging Diagnosis

**DOI:** 10.4317/jced.61925

**Published:** 2024-09-01

**Authors:** Luis Ortiz-Peces, María Álvaro-Martínez, Marta de Uribe-Viloria, Martín Andura-Correas, Eduardo Vázquez-Salgueiro, José Luis Cebrián-Carretero

**Affiliations:** 1 Medical Resident. Oral and Maxillofacial Surgery Department. Hospital Universitario La Paz. Madrid, Spain

## Abstract

Epithelioid hemangioendothelioma (EHE) is a rare neoplasm derived from the vascular endothelium. Although it can occur anywhere in the body, few cases have been described in the oral cavity. We report a 47-year-old woman presenting with a painful ulcerated lesion on the mandibular gingiva, suggestive of a traumatic decubitus ulcer. Histology and immunohistochemistry confirmed the diagnosis of epithelioid hemangioendothelioma. A literature review of EHE of mandibular gingiva was done. Pubmed were searched from 1975 through June 2024 using the following search terms: epithelioid hemangioentothelioma, vascular tumor, oral cavity and mandibular gingiva. Relevant manuscripts were selected and the results were used to update a narrative overview of the diagnosis and management of this entity. We found 38 cases of EHE in the oral cavity, of which 16 were located on the gingiva. Most of them were located on the mandibular gingiva as painless swelling, unlike our case. 70 % of the cases presenting in the mandibular gingiva had bone resorption on imaging. However, only half of those located in the maxillary gingiva had this bone resorption. Only 2 cases located in the mandibular gingiva presented recurrence and 1 of them debuted with nodal metastases after a 7-year follow-up. The clinical and histological diagnosis of EHE is complex and must be confirmed by immunohistochemistry. Upon diagnosing this entity, we should perform an excision with clear margins and conduct long-term follow-up due to the risk of local and distant recurrence.

** Key words:**Epithelioid hemangioendothelioma, Gingival pathologies, Oral cavity, Mandibular Diseases, CD31, Immmunohistochemical markers, Vascular tumor.

## Introduction

Epithelioid hemangioendothelioma (EHE) is a rare vascular neoplasm characterized by endothelial cells with epithelioid or histiocytoid features. Histologically, it is an angiocentric neoplasm characterized by the proliferation of endothelial cells with epithelioid or histiocytoid morphology, featuring vacuolated cytoplasm, and occasionally presenting spindle-shaped cells (1). It is defined by recurrent WWTR1-CAMTA1 or YAP1-TFE3 gene fusion (2,3). Classically, the term “hemangioendothelioma” is used to define those endothelial neoplasms with intermediate malignancy, which today remains a controversial topic. According to the latest WHO classification of soft tissue tumors from 2020, five types of hemangioendothelioma are defined with different clinicopathological characteristics (4). Unlike epithelial neoplasms, which typically dichotomize as either “benign” and “malignant,” this type of mesenchymal neoplasm includes two additional categories: “intermediate (locally aggressive)” and “intermediate (rarely metastasizing)” (5). The EHE variant can appear anywhere in the body, giving lymphatic metastases and in distant organs (mainly pulmonary) in up to 20-30% of patients. It accounts for the cause of death through disease in 10-20% of cases. (6). For this reason, it has been suggested that EHE should be classified as a purely malignant neoplasm along with angiosarcoma (4-7).

Soft tissue EHE is rarely found in the oral cavity. It predominantly affects the extremities, being less frequent its appearance in the head and neck territory (7,8). There are only 38 cases affecting the oral cavity described in the English literature between 1975-2024.

This article focuses its interest on this neoplasm which, in addition to having a malignant behavior, can pose a diagnostic challenge when the primary lesion appears in the attached gingiva. Through the report of a case of HSE of appearance in the mandibular gingiva, we review the literature and discuss the clinical-pathological particularities to consider for the diagnosis, treatment and follow-up of this rare entity.

## Case Report

**Figure 1:**
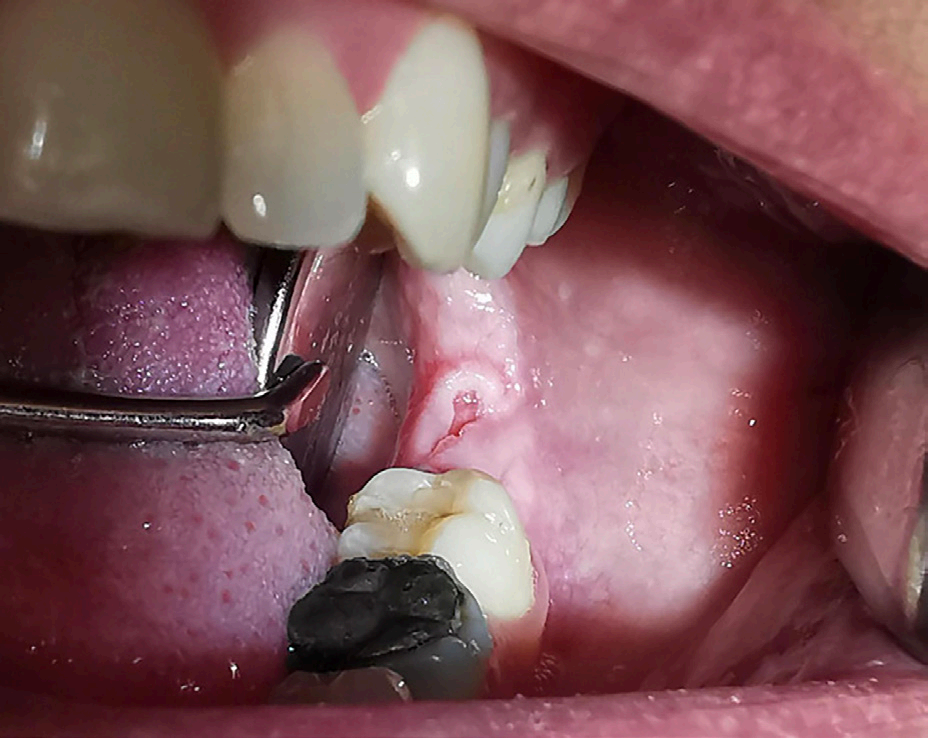
Clinical image of the lesion: pink-colored, ulcerated, 0.6 cm in size in the left retromolar trigone, with raised borders, painful, and non-erythematous mucosa.

**Figure 2:**
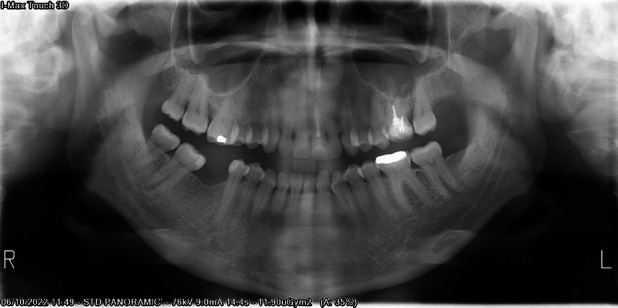
Panoramic radiography (performed after the extraction of tooth 28). No radiolucent images or increase in radiological density are observed in the posterior sector of the third mandibular quadrant.

A 47-year-old caucasian female patient was referred by her dentist to oral and maxillofacial surgeon to evaluate a painful, pink-colored, ulcerated lesion, 0.6 cm in diameter, with raised borders on the gingival mucosa of the left mandibular third molar region, with a 1-month evolution (Fig. 1). The dentist performed the extraction of tooth 28 in case it could be a pressure ulcer, without resolution or morphological changes of the lesion, with a follow-up of one month. The patient had no personal history of systemic diseases or drug use. Clinically, he complained of pain with meals in the area of the injury. A panoramic radiograph showed no radiological signs of involvement of the bone cortical adjacent to the lesion (Fig. 2).

We took an excisional biopsy under local anesthesia and primary closure of the biopsy. Intraoperatively, the specimen we sent for analysis was a spindle of the lesion with a soft rubbery consistency, non-friable.

**Figure 3:**
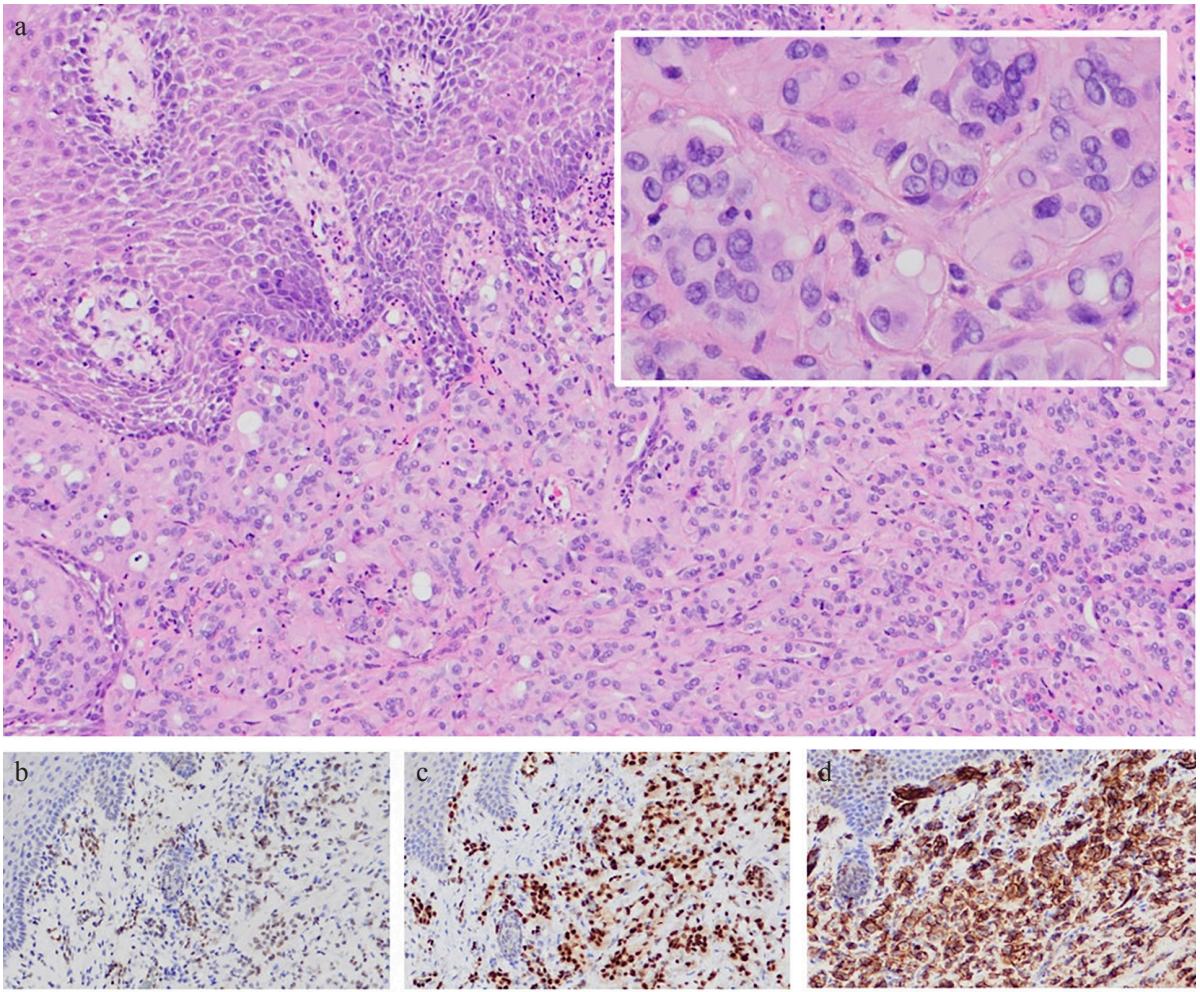
a) Hematoxylin–eosin staining (HE 10x): Nests of polygonal cells set in a collagenous stroma. On higher magnification (HE 60x), intracytoplasmic vacuoles were observed. b) Nuclear expression with FLI1 immunostaining (20x). c) Nuclear expression with ERG immunostaining (20x). d) Membranous expression with CD31 immunostaining (20x).

Microscopic examination showed a squamous mucosa infiltrated by a tumor composed of nests of polygonal cells with abundant eosinophilic cytoplasm and intracytoplasmic lumina. Around some nests, hyalinized collagen was observed. There was no significant cellular atypia, spindle cells, or necrosis (Fig. 3). No mitosis Figures were identified. With ki67, the rate of cell proliferation was approximately 5%. Tumor cells showed positive immunoreactivity to CD31, ERG, FLI1 and negative to CK AE1/AE3, CK7, p63, p40, CD34, S100, HMB45, MelanA, DOG1, mamoglobin, CD68. Epitheliod angiosarcoma was ruled out due to the lack of aggresive histomorphological features. Negativity for S100 and melanic markers excluded a melanoma diagnosis, and the lack of citokeratin expression discarded a carcinoma diagnosis. As an annotation from the anatomical pathology laboratory, they indicated that the orientation of the cut did not allow for an adequate assessment of the surgical margin. However, the lesion seemed to contact it.

**Figure 4:**
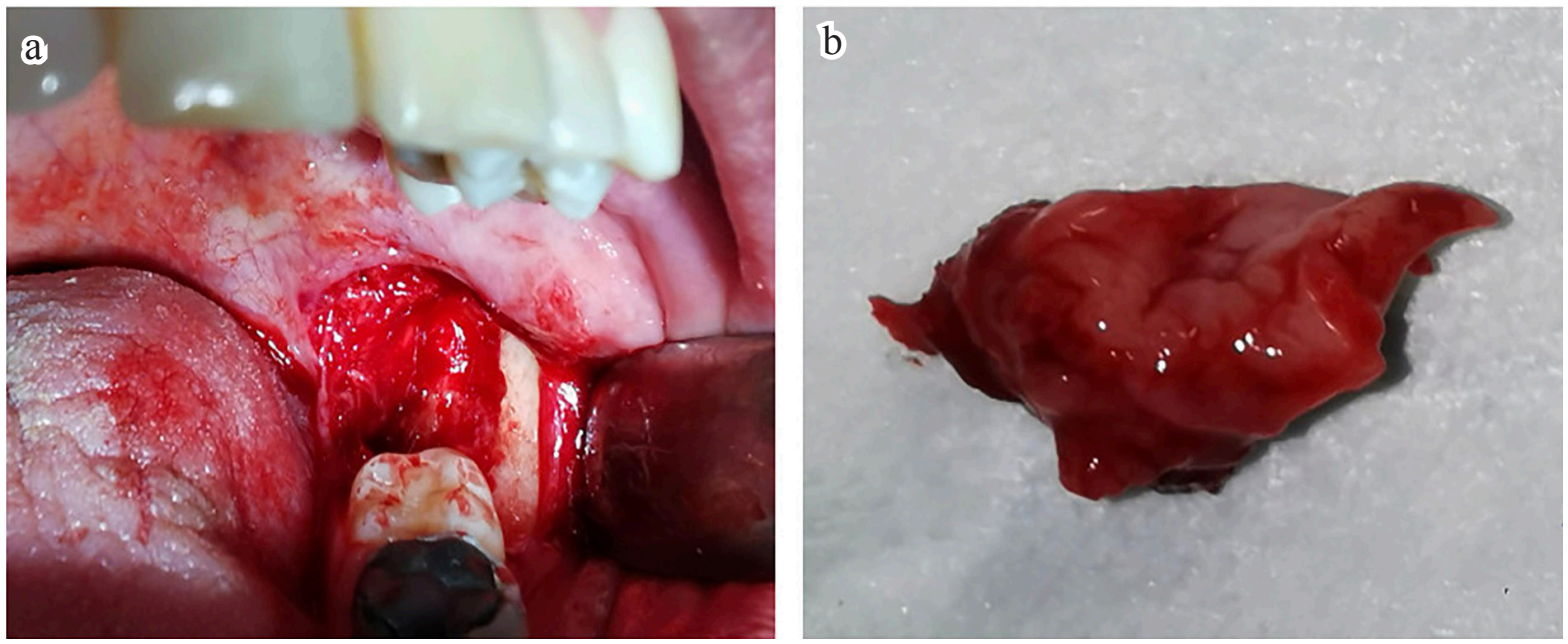
a) Surgical site of margin enlargement with excision of the crestal mucosa of the left retromolar trigone and adjacent mandibular alveolar bone ridge. b) Surgical specimen of enlargement of lesion margins histologically reported as EHE.

The patient was scheduled for surgical margin enlargement. Under locoregional anesthesia, the mucosa of the retromolar trigone was removed along with the adjacent mandibular alveolar bony ridge, hemostasis with oxidized cellulose (SURGICEL®), and primary closure (Fig. 4). Microscopic examination of the specimen was reported as residual epithelioid hemangioendothelioma, 1.5 mm larger in diameter. Surgical edges were negative, with no signs of bone infiltration. The 18-month follow-up with clinical examinations every 3 months and serial panoramic radiographs every 6 months revealed no evidence of tumor recurrence.

## Discussion

EHE can appear in patients of any age, but they are quite rare in children, with a special predilection for the female sex (1,5). However, among the cases with onset in the gingiva collected in our review, half of them occurred in patients under 18 years old, and the majority in females ( Table 1).

Clinically, diagnosis poses a real challenge for medical or dental professionals. EHE that debuts in the gingiva as a primary lesion can be clinically diagnosed as a benign entity. Pyogenic granuloma, giant cell granuloma, fibroma, inflammatory fibrous hyperplasia, or necrotizing ulcerative gingivitis are some of the initial diagnoses that have been considered in lesions that were confirmed as EHE (8,17). This results in a delay in the diagnosis of an entity recognized as a malignant lesion (4,5,8). In the present case, as it began as an ulcerated lesion located in the mandibular gingiva at the occlusal contact of tooth 28, the dentist reasonably thought that it could be a pressure ulcer. Our recommendation is to biopsy those intraoral lesions that have not resolved within a follow-up period of two weeks or longer.

 The Table shows the epidemiological, clinical, radiological and follow-up characteristics of 16 cases of HSE reported in the English literature of presentation in the maxillary or mandibular gingiva ( Table 1). The most common form of intraoral EHE is as a painless pink or reddish swelling. This case has the particularity that the patient reported pain in the location of the lesion, related to meals, probably due to the trauma caused by the upper wisdom tooth in occlusion. Only one case of localization in the mandibular gingiva, published by Sun *et al*. (15), was reported as a painful lesion before. Marrogi *et al*. (12), also published a case of an EHE with painful presentation on the tongue.

More than half of the cases of gingiva localization affected the mandible. Dental mobility was present in 43.75% of the cases of EHE located in the gingiva. 56.25 % of cases showed bone resorption signs on OPG, CBCT or facial CT images. Among the 16 cases presenting in the gums, only 4 recurrences were reported: 3 were local recurrences and 1 lymph node metastasis ( Table 1). However, we consider that the follow-up time is insufficient in most of the reported cases, because recurrences have been reported after a disease-free period of 7 years, as is the case of Ali *et al*. (18).

The confirmatory diagnosis of the lesion is pathological. The differential diagnosis must be established with vascular neoplasms such as angiosarcoma, or even with squamous cell carcinoma. According to the review, the immunohistochemical analysis of the lesion is especially important for establishing a definitive diagnosis (1,5,8). Immunohistochemical positivity for CD34, CD31, factor VIII-R Ag and vimentin suggest that the lesion has its origin in the endothelial epithelium (8). Our case presented negativity for cytokeratin markers, which allows us to rule out the diagnosis of squamous cell carcinoma.

Once EHE has been histologically confirmed with an incisional biopsy, the treatment of choice is complete excision with margins (8,10,14,17,19). In the present case, we excised the lesion with wide margins, along with part of the adjacent alveolar bony ridge. Oxidized cellulose was applied to the surgical site and a direct closure was made. We dismiss the application of platelet-rich plasma due to its relationship with the induction of cell proliferation (21,22).

Our patient is disease-free after a 2-year follow-up. In the present review, after 2 years of follow-up, only 12.5% of cases reported recurrence. However, there are cases of recurrences of EHE after follow-up of up to 8 years (18,23).

## Conclusions

EHE has a malignant behavior with a risk of metastasis. Its location in the gingiva can mimic multiple benign entities that delay diagnosis. Histological study of EHE can give an erroneous diagnosis, therefore immunohistochemical analysis is mandatory to confirm the diagnosis. Wide resection of the lesion with margins and long-term follow-up of the patient is recommended.

## Figures and Tables

**Table 1:** Clinical and radiographic features for cases of gingival EHE reported in English litererature.

Author (year)	Age	Sex	Location	Clinical features	Bone resorption	Recurrence	Follow up
Wesley et al. (1975) (9)	18	F	Mandibular	Reddish erosive lesion, (34 to 36)	Yes	No	2 years
Ellis, Kratochvil (1986) (10)	13	F	Maxillary	Swelling, pink, tooth mobility	No	No	6 years
Ellis, Kratochvil (1986) (10)	4	F	Mandibular	Tooth mobility	Yes	NOR	NOR
de Araujo et al. (1987) (11)	4	M	Mandibular	Swelling, ulceration, tooth mobility	No	NOR	NOR
Marrogi et al. (1991) (12)	36	M	Maxillary	Erythematous lesion, 1.5 cm	No	Yes	6 months (until recurrence)
Flaitz et al. (1995) (13)	7	F	Mandibular	Reddish swelling, 1.5 cm, tooth mobility	Yes	No	52 months
Chi et al. (2005) (14)	28	F	Maxillary	Purple swelling, 0.6 cm	No	No	8 months
Sun et al. (2007) (15)	12	M	Maxillary	Ulcerated swelling, 3.0 cm, tooth mobility	Yes	No	6 months
Sun et al. (2007) (15)	11	M	Mandibular	Painful swelling, 2.0 cm, tooth mobility	Yes	No	8 years
Mohtasham et al. (2008) (16)	9	M	Maxillary	Ulcerated reddish swelling	No	Yes	1 year (until recurrence)
Gordón-Núñez et al. (2008) (8)	17	F	Mandibular	Pink, exophytic, swelling, 2.0 cm	No	No	14 months
Salgarelli et al. (2014) (17)	32	M	Mandibular	Soft mucosal formation, gingival recession, 1.5 cm	Yes	Yes (NM)	4 years (until recurrence)
Ali et al. (2015) (18)	23	F	Mandibular	Erythematous swelling, tooth mobility	Yes	Yes	7 years (until recurrence), 21 years later free of lesion
Rajendrakumar et al. (2019) (19)	41	F	Maxillary and mandibular	Reddish, painless, ulcerated, friable Maxillary: 3 x 4 cm Mandibular: 1 x 2 cm	Yes, both	NOR	NOR
Januzis et al. (2020) (20)	18	F	Maxillary	Gingival recession, ulcerated, painless. 1. 3 cm	Yes	No	31 months
Present article (2024)	47	F	Mandibular	Painful, pink, ulcerated, swelling, 0.6 cm	No	No	18 months

M: Male; F: Female; NI: No information; NM: Nodal Metastases; NOR: No reference.

## Data Availability

The datasets used and/or analyzed during the current study are available from the corresponding author.
